# Sex Differences in Age-Associated Type 2 Diabetes in Rats—Role of Estrogens and Oxidative Stress

**DOI:** 10.1155/2019/6734836

**Published:** 2019-04-07

**Authors:** Ana Díaz, Raúl López-Grueso, Juan Gambini, Daniel Monleón, Cristina Mas-Bargues, Kheira M. Abdelaziz, José Viña, Consuelo Borrás

**Affiliations:** ^1^UCIM, Universitat de València, Valencia, Spain; ^2^Freshage Research Group-Department of Physiology, Faculty of Medicine, University of Valencia, CIBERFES, INCLIVA, Avenida Blasco Ibañez n° 15, 46010 Valencia, Spain; ^3^Department of Pathology, Faculty of Medicine, University of Valencia, CIBERFES, Metabolomic and Molecular Image Laboratory, Fundación Investigatión Clínico de Valencia (INCLIVA), Valencia, Spain

## Abstract

Females live longer than males, and the estrogens are one of the reasons for this difference. We reported some years ago that estrogens are able to protect rats against oxidative stress, by inducing antioxidant genes. Type 2 diabetes is an age-associated disease in which oxidative stress is involved, and moreover, some studies show that the prevalence is higher in men than in women, and therefore there are sex-associated differences. Thus, the aim of this study was to evaluate the role of estrogens in protecting against oxidative stress in type 2 diabetic males and females. For this purpose, we used Goto-Kakizaki rats, which develop type 2 diabetes with age. We found that female diabetic rats showed lower glycaemia levels with age than did diabetic males and that estrogens enhanced insulin sensitivity in diabetic females. Moreover, glucose uptake, measured by positron emission tomography, was higher in the female brain, cerebellum, and heart than in those from male diabetic rats. There were also sex-associated differences in the plasma metabolic profile as determined by metabolomics. The metabolic profile was similar between estrogen-replaced and control diabetic rats and different from ovariectomized diabetic rats. Oxidative stress is involved in these differences. We showed that hepatic mitochondria from females produced less hydrogen peroxide levels and exhibited lower xanthine oxidase activity. We also found that hepatic mitochondrial glutathione oxidation and lipid oxidation levels were lower in diabetic females when compared with diabetic males. Ovariectomy induced oxidative stress, and estrogen replacement therapy prevented it. These findings provide evidence for estrogen beneficial effects in type 2 diabetes and should be considered when prescribing estrogen replacement therapy to menopausal women.

## 1. Introduction

Diabetes mellitus is a chronic disease of high prevalence. According to the World Health Organization, there are currently approximately 143 million people with diabetes worldwide. This figure is expected to rise to 300 million by the year 2025 due, above all, to the increase, aging, and urbanization of the population [1]. It is estimated that its prevalence is around 5%, increasing significantly in relation to age: it reaches figures between 10 and 15% in the population over 65 years of age and 20% if we consider only those older than 65 to 80 years [2]. This happens because aging leads to an increase in fasting plasma glucose levels of 1 to 2 mg % per year and postprandial glycaemia increases from 8 to 20 mg %. These changes would be linked to an alteration of the peripheral or hepatic sensitivity to insulin or to an alteration of pancreatic islet function [3]. Of the two basic forms of diabetes, type 2 occurs mainly in adults and is, with much, the most common form. It represents between 85 and 90% of all cases of diabetes [1]. There are sex-specific differences in the prevalence of type 2 diabetes, and it is lower in women compared with men [4]. However, when women reach menopause, the risk of developing diabetes (type 2) increases to levels between 6 and 20% [5]. Type 2 diabetes is caused by the combination of a decrease in the effect of insulin acting in the body, associated with the inability of the *β*-pancreatic cells to produce adequate amounts of insulin. It has been shown that estradiol exerts favorable effects on insulin and glucose metabolism *in vivo*, such as increase in pancreatic insulin secretion [6], improvement of peripheral sensitivity to insulin [7], reduction of blood glucose and basal insulinemia [8], increased afterload blood glucose [9], improvement of the lipid effects of insulin [10], prevention of *β*-pancreatic cell apoptosis [11], and prevention of obesity [12].

After menopause, pancreatic insulin secretion decreases and insulin resistance increases, changes that may be due to the combination of aging [13] and deficiency of estrogen. This deficiency also affects the blood flow to the muscle further limiting the already reduced glucose intake. Currently, clinical studies have been conducted to assess the effect of hormone replacement therapy (HRT) on the development of diabetes disease [14–16]. The following conclusions are derived from them: administration of estrogen combined with progestin (E + G) reduces the risk of developing diabetes by 21% compared to placebo. After one year of treatment, E + G slightly reduces blood glucose and insulin levels, which is probably the reason why the number of diabetes is minor in this group. Although E + G slightly reduces body weight and waist circumference in women after one year, this factor has no influence on the development or not of diabetes.

Projections made indicate that by 2020, approximately 124 million of people will live more than 80 years and the majority of that population will be made up of menopausal women, so women will live more than a third of their lives with estrogen deficiency. The study of the role of estrogen in the development of diabetes is therefore of great importance.

It has already been mentioned that Type 2 diabetes is associated with aging, and we must mention that it is also associated with oxidative stress: glucose is prone to oxidation even under physiological conditions [17, 18]. It has also been shown that the activity of xanthine oxidase, an enzyme that generates superoxide radical, increases in diabetic rats (type I diabetes) [19, 20]. The antioxidant role of estrogens has been amply demonstrated [21]. We studied the mechanisms by which females live longer than males and demonstrate the role of the different rates of free radical production in this process [22–26]. We also observed that estradiol was responsible for this protection against oxidative stress [27–29]. Given that there is a considerable difference in the prevalence of diabetes mellitus between men and women and that this is also related to oxidative stress, our aim was to study the role of oxidative stress in the different prevalence of this disease between genders and to determine the possible protective role of estrogens, as well as the mechanisms by which this possible action exerts. This would allow a deeper knowledge about the effects of HRT on diabetes, since few authors have related the protective role of estrogen in diabetes due to its antioxidant effect [9, 11, 30]. In addition, we cannot fail to mention the complications associated with diabetes at various levels (eye disease, kidney, neuropathy, and cardiovascular). Regarding the relationship between diabetes and cardiovascular disease, this is the main cause of diabetes-related deaths, so that mortality from this disease in adults is 2 (in men) to 4 (in women) greater than mortality in adults without diabetes [31]. This worse prognosis of diabetic women is correlated with an increase in oxidative stress and decreased antioxidant defenses, and these are postulated as a cause probable of the greater susceptibility in the female sex to cardiovascular complications, with more frequent and diffuse coronary lesions. So, the study of oxidative stress in diabetes, and specifically its importance in the difference between males and females, is of great interest.

## 2. Material and Methods

### 2.1. Experimental Animals

Male and female Wistar and Goto-Kakizaki (GK) rats were used. GK rats are an excellent model of type 2 diabetes, which also does not present obesity like other models. In this way, we can rule out obesity as experiment variable. They also show similar metabolic, hormonal, and vascular disorders than in human diabetes. Its characteristics include hyperglycemia, defects in the secretion of insulin in response to glucose both *in vivo* and in isolated pancreatic cells, and insulin resistance, both hepatic and peripheral. Complications derived from the diabetes that develops this model are retinopathy, microangiopathy, neuropathy, and nephropathy. The control of the diabetization was carried out through clinical observation (polydipsia, polyphagia, and thinning) and by determining biochemical parameters as the measurement of fasting glucose, using a glucosimeter (Accu-Chek Aviva de Roche). We established an endpoint criterion for the sacrifice of rats with 200 mg/dL of blood glucose. Thus, the GK males were sacrificed at 11-15 months of age and the GK females at 20-22 months of age. As Wistar controls, we used rats of similar ages to those corresponding to their sex.

Rats were stored at the animal house of the Faculty of Medicine of the University of Valencia. Handling, supervision, and experimentation with rats were done in accordance to the recommendations of the Federation of European Laboratory Animal Science Associations. All the work complies with both national and EU legislation—Spanish Royal Decree RD 1201/2005 and EU Directive 86/609/CEE as modified by 2003/65/CE—for the protection of animals used for research experimentation and other scientific purposes. All protocols were previously subjected and approved by the Ethical Committee of the University of Valencia.

Animals were housed at constant temperature and humidity and with a 12 h light/12 h dark cycle. They were fed on a standard laboratory diet (containing 590 g carbohydrates, 30 g lipids, and 160 g protein per kilogram of diet) and tap water *ad libitum*.

### 2.2. Ovariectomy Procedure

Ovariectomy was performed as follows: Rats were anesthetized with ketamine (100 mg/kg) and acepromazine (2.5 mg/kg). Abdominal skin was cut, the peritoneum was opened, both ovarian arteries were ligated, and both ovaries were removed. Sham-operated rats underwent the same procedure, but ovarian arteries were not ligated and ovaries were not removed. Animals were allowed to recover for at least three weeks before experiments were performed. Estrogen levels after ovariectomy fall to about 10% of the controls [32]. Where indicated, an estrogen replacement therapy of 17*β*-estradiol was given subcutaneously at a daily dose of 1 *μ*g/kg body weight for three weeks. This results in an estrogen level similar to controls. This dose was chosen because it was shown that this estrogen dose does not cause changes in liver weight or increases in DNA content [33].

Female rats we divided into 3 groups: sham-operated (Sham), ovariectomized for 3 weeks (OVX), and ovariectomized for 3 weeks and immediately replaced with estradiol (OVX+E).

We have used different tissues in our studies. We measured radical production and antioxidants in liver because we have previously done so in basic experiments trying to understand why females live longer than males and why they produce lower oxidant production, and we did this in the liver because mitochondria are much more uniform than those of the brain. This is the reason for our studies in antioxidant production in liver. However, when trying to measure glucose uptake, the brain is a much better tissue. The liver is characterized (classical as well as PET studies confirm this) by changing the glucose output dramatically from one situation to another. For instance, the liver can take up glucose or release it, depending on the particular metabolic moment of the animal. Thus, the liver (together with the kidney cortex) is the only tissue that can either release glucose or take it up. Glucose uptake by the liver is low, and the PET signal is very low. This contrasts sharply with the case of the brain. Therefore, the brain was a better choice to measure glucose uptake. We also determined heart glucose uptake for the importance of cardiovascular disorders in type 2 diabetes.

Finally, metabolomics is usually performed in serum/plasma. The reason for using plasma is twofold: firstly, this is the milieu used for human studies for obvious ethical reasons, and secondly, plasma gives a general view of the metabolic situation of the animal.

### 2.3. Glucose Tolerance Test

We kept our rats in a 6-hour fast and took a sample of the basal glycaemia (time 0); after this, they were given a dose of 2 g glucose/kg live weight orally by an esophageal tube (18 G-40 mm). We subsequently carried out glycaemia measurements at times 15, 30, 60, 120, and 180 minutes of basal. The blood samples were obtained from the saphenous vein, according to the method described above, and blood glucose was measured using the glucose meter (Accu-Chek revives the Roche brand).

### 2.4. Determination of Brain and Heart Glucose Uptake *In Vivo*

Rats were deprived of food for 8–14 h before 18F-2-fluor-2-deoxiglucose (18F-FDG) injection. 18F-FDG (5.8–11.1 MBq) was injected intraperitoneally after anesthesia with isoflurane (1.5–2% in 100% oxygen, IsoFlo; Abbott Laboratories). PET was started 60 min after 18F-FDG injection as described in [34]. 18F-FDG was synthesized as previously described [35]. The administered dose (FDG activity) was indeed corrected for body weight. We acquired 20 min static images 60 min after injection of 18F-FDG. The biodistribution of 18F-FDG by the heart was compared between all the studied groups. The PET images were obtained with the ALBIRA small animal PET (ONCOVISION, GEM Imaging). Regions of interest were manually drawn over the brain with PMOD software. Tracer uptake by heart was quantified as SUV (Standardized Uptake Value, Total Sum) with a methodology employed before in [36].

### 2.5. Storage, Preparation, and ^1^H NMR Spectroscopic Analysis of Blood Plasma

Rats' blood plasmas were stored at -80C and thawed before use. For NMR analysis, as shown in [36], 20 *μ*L of plasma was mixed with 2 *μ*L of D_2_O (as a field lock). A total of 20 *μ*L of the mixture of each sample was individually transferred into a 1 mm high-quality NMR capillary. All ^1^H NMR spectra were acquired using a standard one-dimensional pulse sequence with water suppression (Bruker Avance 600 spectrometer operating at 600.13 MHz with a 1 mm ^1^H/^13^C/^15^N TXI probe). A total of 256 FIDs, free induction decay, were collected into 64 k data points with a spectral width of 14 ppm and a recycle delay (RD) of 1 s. Water signal was saturated with a weak irradiation during the recycle delay. Before Fourier transformation, the FID was multiplied by a 0.3 Hz exponential line broadening. Spectral chemical shift referencing on the alanine CH_3_ doublet signal at 1.475 ppm was performed in all spectra. Spectral regions between 0.5 and 4.5 ppm, and between 5.5 and 9.5 ppm, were binned in segments of 0.01 ppm width (6 Hz) for multivariate analysis. We normalized the binned data to the total spectral area. Available spectral databases and 2D NMR experiments were used to aid structural identification of relevant metabolites. Finally, using in-house scripts for data analysis, all spectra were processed using Mnova (MestreLab, Santiago de Compostela, Spain) and transferred to MATLAB® (MathWorks Inc., 2006) as shown in [36].

### 2.6. Multivariate Analysis of NMR Spectra

In multivariate data, as explained in [36], the representation of any two variables against each other is not sufficient to obtain global correlations. Principal component analysis (PCA) is a method for low-dimensional representation of multivariate data, which optimally preserves the structure of the data. PCA produces a transformation of the variables in a data set into new latent variables called principal components (PCs). These PCs do not correlate to each other and explain decreasing proportions of the total variance of the original variables. Considering that larger variances express larger amounts of information, a compact description of the data sets and the relationships between samples can be generated. Scores for each sample are calculated in these new PCs and plotted in score plots. The loadings of the different PCs help in determining which variables contain more information [37]. We used PLS_Toolbox 6.7 (Eigenvector Research, WA, USA) for MATLAB® to build the PCA models. The PCA model was cross-validated by the leave-one-out method providing a cross-validation RMS of 0.17. Hotelling's T2 for 95% interval of confidence was 12.15.

### 2.7. Isolation of Mitochondria

Animals were sacrificed by cervical dislocation, and then their livers were quickly removed. Mitochondria from the liver were obtained by differential centrifugation, as described by Rickwood et al. [38].

### 2.8. Peroxide Production Determination

The rate of peroxide production was determined using a modification of the method described by Barja [39] in isolated mitochondria. Concisely, we incubated the mitochondria at 37°C with 10 mM succinate in 2 mL of phosphate buffer, pH 7.4, containing 5 mM KH_2_PO_4_, 0.1 mM EGTA, 3 mM MgCl_2_, 30 mM HEPES, 145 mM KCl, 0.1 mM homovanillic acid, and 6 U/mL horseradish peroxidase. The incubation was stopped at 5, 10, and 15 min with 1 mL of cold 2 M glycine buffer containing 2.2 M NaOH and 50 mM EDTA. Supernatant fluorescence was measured using 312 nm as excitation wavelength and 420 nm as emission wavelength. The peroxide production rate was then calculated using a standard curve of H_2_O_2_ [40].

### 2.9. Xanthine Oxidase Determination

XO activity was measured in plasma and soleus muscle by the fluorimetric method described in Beckman et al. [41]. Briefly, isoxanthopterin formation from pterine was followed fluorometrically as previously described (excitation at 345 nm and emission at 390 nm) [42, 43].

### 2.10. Measurement of Oxidative Stress Parameters

Lipid peroxidation was determined as accumulation of MDA, which was detected by HPLC as an MDA–thiobarbituric acid adduct [44].

Determination of GSH and GSSG was carried out using the high-performance liquid chromatography method, with UV–visible detection, which we developed to measure GSSG in the presence of a large excess of GSH [18, 45]. The essence of this method consists of minimizing GSH oxidation, which then would result in a large increase in GSSG. To measure GSH, isolated mitochondria were treated at 4°C with 12% ice-cold perchloric acid containing 2 mM bathophenanthroline disulfonic acid (BPDS). For GSSG determination, isolated mitochondria were treated at 4°C with 12% ice-cold perchloric acid containing 2 mM BPDS (Sigma Chemical Co.) and 40 mM N-ethylmaleimide (Sigma Chemical Co., St. Louis, MO, USA), in order to prevent GSH oxidation. Samples were then centrifuged at 15,000*g* for 15 min at 4°C, and we used the acidic supernatants for total glutathione and GSSG measurements.

### 2.11. Statistical Analysis

Data were represented by mean ± standard deviation (SD). Normality of distribution was checked with the Shapiro–Wilk test, and homogeneity of variance was tested by Levene's statistics. Comparison between groups was performed with a one-way ANOVA and two-tailed *t*-test. *P* values <0.05 were considered statistically significant.

## 3. Results and Discussion

### 3.1. Males Are More Prone to Develop Type 2 Diabetes with Age than Females Are—Role of Estrogen Replacement Therapy

**Figure 1:**
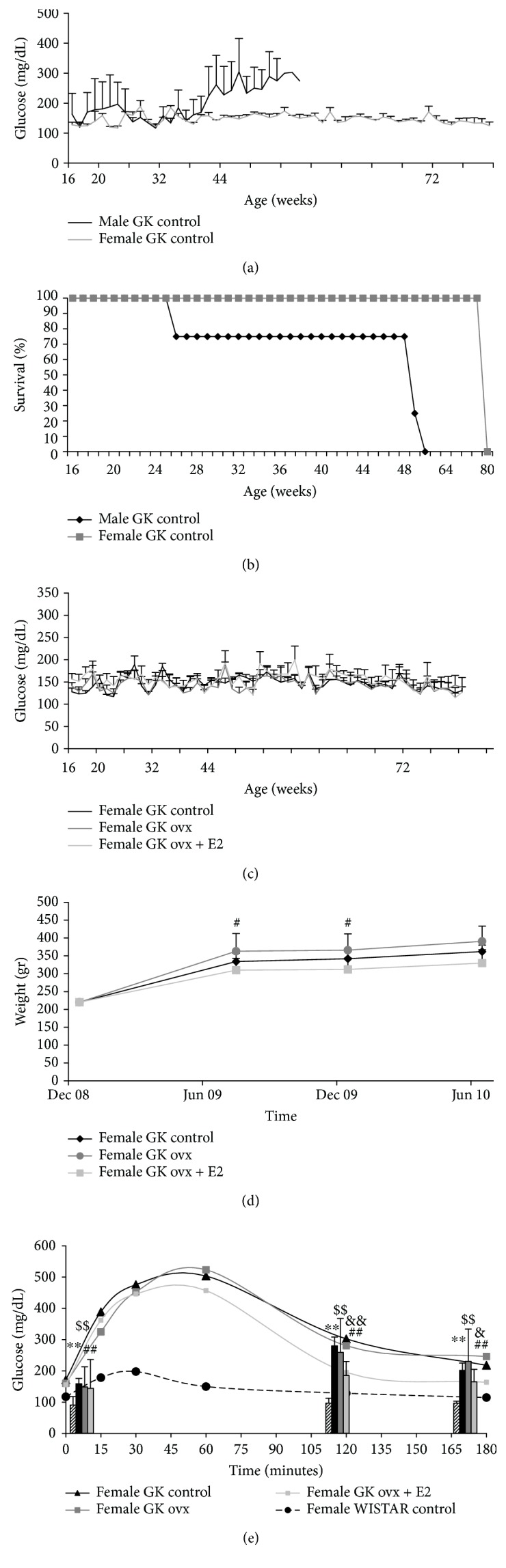
Males are more prone to develop type 2 diabetes with age than females. Role of estrogen replacement therapy. (a) Evolution of glycaemia levels in male and female GK controls. The data are shown as means ± SD (*n* = 4 for both groups). The statistical significance is expressed as ^∗^*p* < 0.05. (b) Survival curve in male and female GK controls (*n* = 4 for both groups). (c) Evolution of glycaemia levels in female GK controls (*n* = 4), ovariectomized female GK (*n* = 6), and ovariectomized female GK + estradiol replacement (*n* = 6). The data are shown as means ± SD. (d) Evolution of weight in female GK controls (*n* = 4), ovariectomized female GK (*n* = 6), and ovariectomized female GK + estradiol replacement (*n* = 6). The data are shown as means ± SD. The statistical significance is expressed as ^#^*p* < 0.05 for GK OVX versus GK OVX + E2. (e) Glucose tolerance test in female WISTAR controls (*n* = 6), female GK controls (*n* = 4), ovariectomized female GK (*n* = 6), and ovariectomized female GK + estradiol replacement (*n* = 6). The data are shown as means ± SD. The statistical significance is expressed as ^∗∗^*p* < 0.01 for WISTAR CTL versus GK CONTROL, ^##^*p* < 0.01 for WISTAR CTL versus GK OVX + E2, ^$$^*p* < 0.01 for WISTAR CTL versus GK OVX, ^&^*p* < 0.05 and ^&&^*p* < 0.01 for GK OVX versus GK OVX + E2.

We first studied the evolution of glycaemia levels in male and female GK controls, and as shown in Figure 1(a), glycaemia levels in males started to increase at 37 weeks of age, but it was not increased in females with age. To deepen this fact, we established an endpoint criterion of glycaemia above 200 mg/dL for sacrificing the rats.  Figure 1(b) shows that female GK rats lived longer than did GK male rats, meaning that males reached the endpoint criteria before females did. Indeed, the half span was 52 weeks for males and 80 for females.

It is very well-known that estrogens protect females against many physio-pathological processes [46–51], and it is even involved in the longer longevity of females versus males [24–26, 40]. Moreover, it has been demonstrated its protective role against diabetes [11, 12, 52–56]. Therefore, we ovariectomized females to check the role of estrogens in protecting females against type 2 diabetes development. We also included a group with estrogen replacement therapy to better demonstrate the protective role of estrogens. When we measured glycaemia level evolution with age, we did not find any difference between the GK groups (control, ovx, and ovx+E2) (see Figure 1(c)). However, as shown in Figure 1(d), we found that ovariectomized GK rats gained more weight with the evolution of the disease than the other two groups did. This is very important as obesity is one of the factors that can worsen the disease and its complications [57, 58]. Moreover, we assessed insulin sensitivity by a glucose tolerance test, and we clearly showed that estrogen replacement therapy prevented the increase in glucose levels shown in control and ovariectomized GK female rats. We also included a nondiabetic group of rats (Wistar rats) as control of the diabetic rats (see Figure 1(e)). The protective role of estrogens against insulin resistance has been widely demonstrated [11, 55, 56, 59–61]. Many of the studies have evaluated the insulin resistance in obese models of diabetes, or in combination with a high-fat diet [55, 62, 63]. In our study, we show that in rats which are not obese and develop type 2 diabetes with age, estradiol is also able to enhance insulin sensitivity, allowing its protective role against this age-associated disease.

### 3.2. Glucose Uptake Is Higher in Female Brain, Cerebellum, and Heart than in Those from Male Diabetic Rats

Insulin resistance is associated with significantly lower regional cerebral glucose metabolism, which in turn may predict worse memory performance [64]. Moreover, several groups have shown that insulin-mediated myocardial glucose uptake is reduced in type 2 diabetic patients in comparison with healthy control subjects [65, 66]. This may play a role in the known cardiovascular differences between men and women with diabetes.

**Figure 2:**
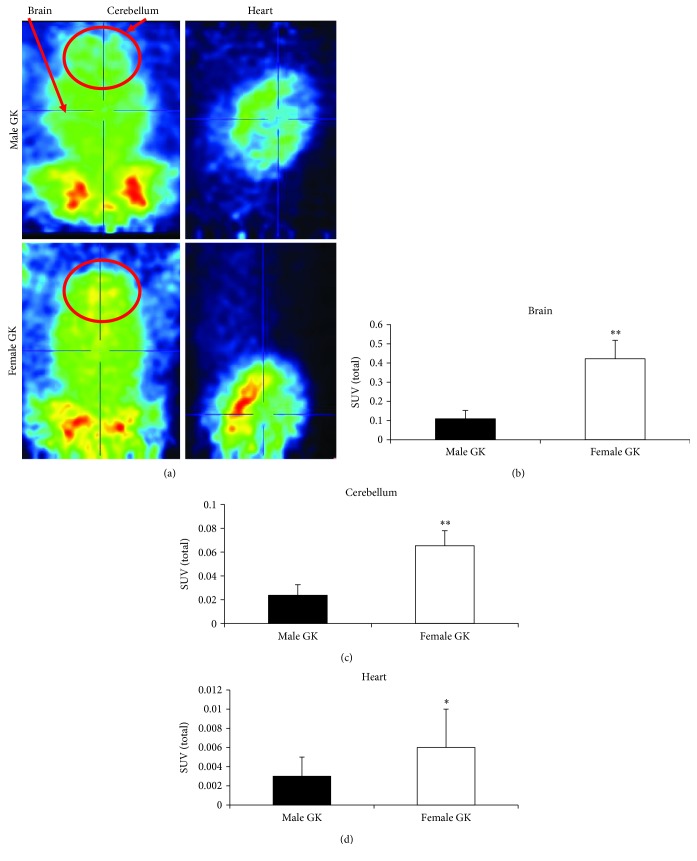
Glucose uptake is higher in the female brain, cerebellum, and heart than in those from male diabetic rats. (a) Representative PET images showing glucose consumption in the brain, cerebellum, and heart in male GK and female GK. (b) Glucose consumption in the brain, (c) cerebellum, and (d) heart in male GK and female GK. The data are shown as means ± SD (*n* = 4 in all cases). The statistical significance is expressed as ^∗∗^*p* < 0.01.

In our model, we determined glucose consumption by PET in different tissues of male and female diabetic rats.  Figure 2(a) show representative PET images of glucose consumption in the brain, cerebellum, and heart demonstrating that in all tissues, it was lower in males than in females. Figures 2(b)–2(d) show the analysis (SUV (standardized uptake value)) of the PET images in the respective tissues, which indicate that there are statistically significant differences between male and female diabetic rats.

Therefore, the diabetic brain and heart from males are characterized by a reduced capacity to uptake glucose and insulin resistance in comparison with those of females.

### 3.3. Metabolic Profile Shows that Type 2 Diabetes Has a Very High Impact in the Metabolism, and There Are Sex Differences—Role of Estrogen Replacement Therapy

Metabolomics offers a unique opportunity to study global metabolic profiles in animals (or persons) in different physiological and pathophysiological situations [67]. The principal component analysis represents scores plot of plasma metabolomic data. Distance between samples in the PCA space represents differences in global metabolic profiles.

**Figure 3:**
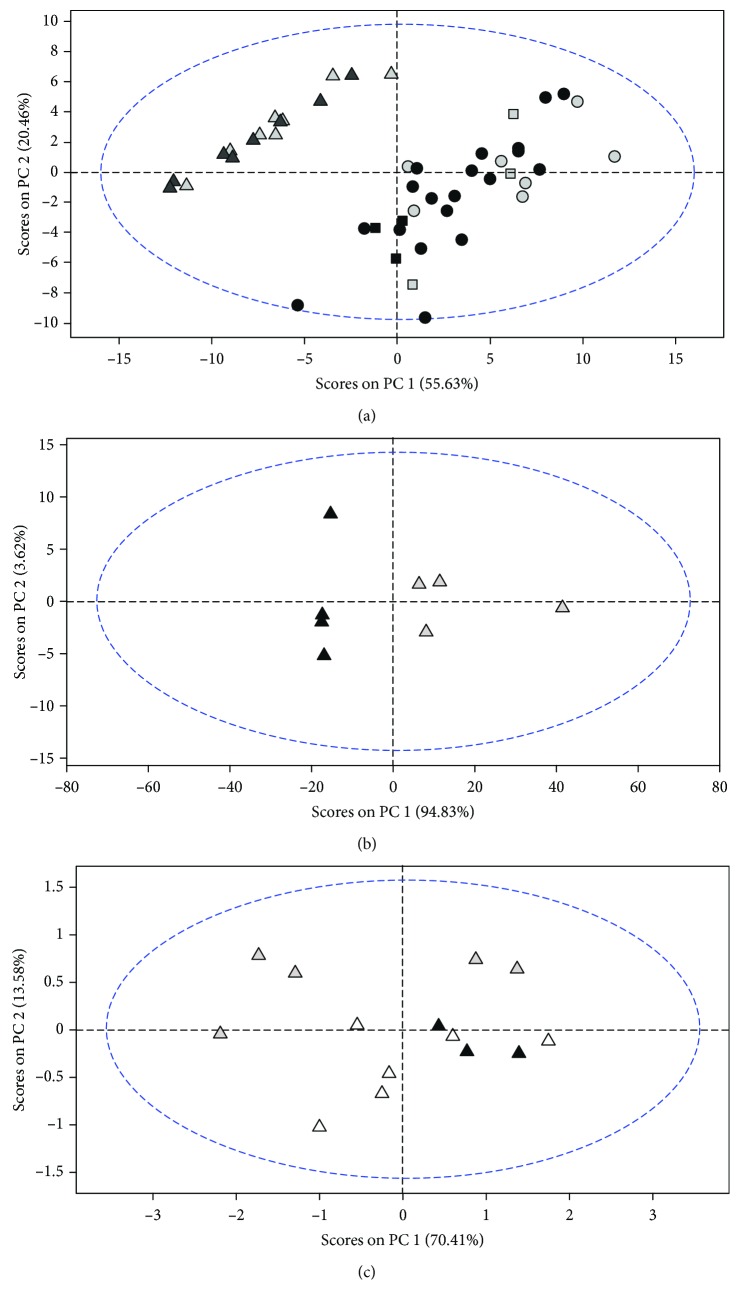
Metabolic profile shows that type 2 diabetes has a very high impact in the metabolism, and there are sex differences. Role of estrogen replacement therapy. (a) Metabolic profile of male GK (black triangle), female GK (gray triangle), young male (black circle), young female (gray circle), old male (black square), and old female (gray square). (b) Comparison of the metabolic profile of male GK (black triangle) and female GK (gray triangle). (c) Comparison of the metabolic profile of female GK controls (dark triangle) (*n* = 3), ovariectomized female GK (gray triangle) (*n* = 6), and ovariectomized female GK + estradiol replacement (white triangle) (*n* = 5).

Figure 3(a) shows that type 2 diabetes has a very high impact in the plasma metabolic profile. Samples from male (black triangle) and female (gray triangle) diabetic rats are well separated in the PCA space, representing a different metabolic profile, to those from young and old control male and female rats (young male (black circle), young female (gray circle), old male (black square), and old female (gray square)). This fact has been previously demonstrated by Knebel et al., who showed that metabolome analyses from diabetic patients enable identification of defined diabetes type-specific differences and detection of biomarkers of insulin sensitivity [68]. When analyzing diabetic rats specifically (Figure 3(b)), we find that there are also sex differences in the metabolic profile. Samples from male diabetic rats (black triangle) are clearly separated from the female diabetic rats (gray triangle), showing a different metabolic profile.

To check the role of estrogens in this difference (see Figure 3(c)), we also determined the metabolic profile in ovariectomized and estrogen-replaced diabetic rats. Samples from ovariectomized rats (gray triangle) are separated in the PCA space in the vertical direction, representing a different metabolic profile, to those from female diabetic rats. On the contrary, rats treated with estrogen replacement therapy (white triangle) are closer in the vertical direction in the PCA space to those of female diabetic rats (black triangle), representing a similar global metabolic profile. Therefore, estrogen replacement therapy restores in part the metabolic profile of female diabetic rats.

Studies performed with diabetic postmenopausal women have shown clear alterations in the lipid metabolic profile and that estrogen replacement therapy can modulate these menopause-associated changes [69]. Mosnier-Pudar et al. conclude that postmenopausal replacement therapy appears preferable in this vascular high-risk type 2 diabetic population, particularly since estrogens may have an antiatherogenic effect by direct action on the vessel walls. However, more studies are needed to establish the possible protective role of estrogen replacement therapy in postmenopausal women suffering from type 2 diabetes [70].

Therefore, we find that type 2 diabetes has a profound impact in the metabolic profile in plasma and that indeed there are sex differences involved, which in part are due to the presence of estrogens.

### 3.4. Type 2 Diabetic Female Rats Are Protected against Free Radical Production Compared with Type 2 Diabetic Male Rats—Protective Role of Estrogen Replacement Therapy

**Figure 4:**
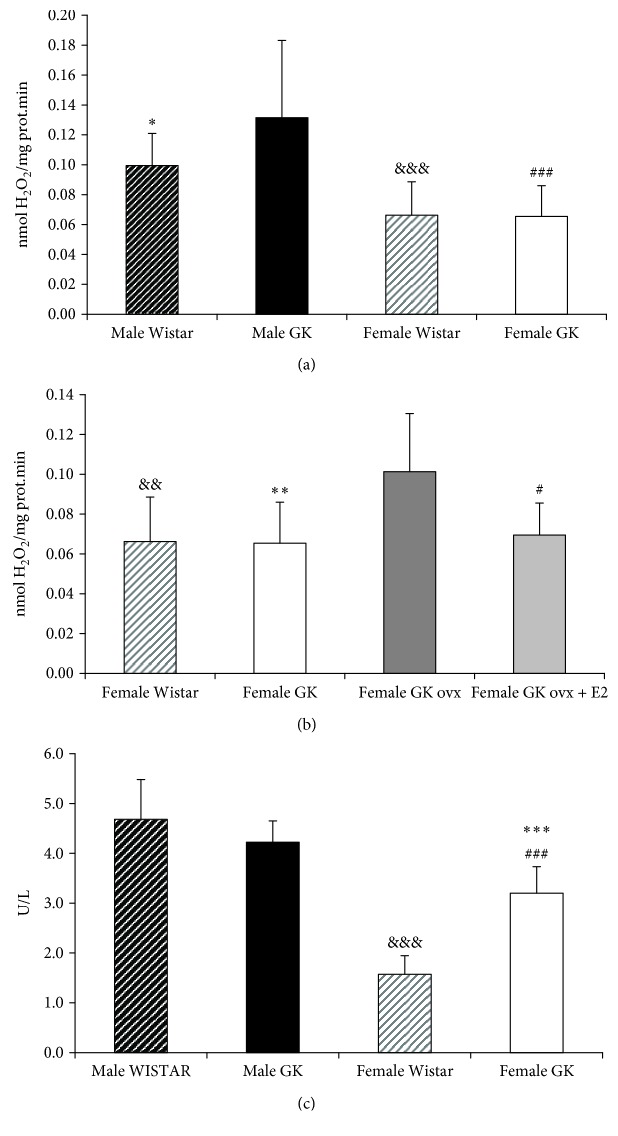
Type 2 diabetic female rats are protected against free radical production compared with type 2 diabetic male rats. Protective role of estrogen replacement therapy. (a) Mitochondrial hydrogen peroxide production in male WISTAR (*n* = 12), male GK (*n* = 8), female WISTAR (*n* = 14), and female GK (*n* = 8). The data are shown as means ± SD. The statistical significance is expressed as ^∗^*p* < 0.05 for male WISTAR versus male GK, ^###^*p* < 0.001 for male GK versus female GK, and ^&&&^*p* < 0.001 for male WISTAR versus female WISTAR. (b) Mitochondrial hydrogen peroxide production in female WISTAR (*n* = 14), female GK (*n* = 4), ovariectomized female GK (*n* = 6), and ovariectomized female GK + estradiol replacement (*n* = 6). The data are shown as means ± SD. The statistical significance is expressed as ^&&^*p* < 0.01 for WISTAR versus GK OVX, ^∗∗^*p* < 0.01 for GK versus GK OVX, and ^#^*p* < 0.05 for GK OVX versus GK OVX + E2. (c) Xanthine oxidase activity levels in plasma of male WISTAR (*n* = 7), male GK (*n* = 8), female WISTAR (*n* = 5), and female GK (*n* = 8). The data are shown as means ± SD. The statistical significance is expressed as ^###^*p* < 0.001 for female GK versus male GK, ^∗∗∗^*p* < 0.001 for female GK versus female WISTAR, and ^&&&^*p* < 0.01 for female WISTAR versus male WISTAR.

Oxidative stress is one of the mechanisms involved in the pathophysiology of type 2 diabetes [71]. Some years ago, we showed that there are sex-associated differences in oxidative stress and that estrogens protect females against its burden [27–29, 40]. This prompted us to study possible sex-associated oxidative stress differences in diabetic rats, which could explain in part the mechanism underlying why females are protected against the disease when compared with males. For this reason, we measured hydrogen peroxide production in hepatic mitochondria from male and female diabetic rats and, as shown in Figure 4(a), it was higher in those of males when compared with females. Moreover, when we compared with nondiabetic rats, we noted that diabetic males produced higher hydrogen peroxide levels than nondiabetic males did, but, on the contrary, diabetic females produce similar levels of peroxides when compared with nondiabetic rats. This means that female diabetic rats were protected against oxidative stress, but males do not. To check again the role of estrogens, we determined hydrogen peroxide production in ovariectomized and estrogen-replaced diabetic rats (Figure 4(b)), and we found that ovariectomized diabetic female rats showed an increased production compared with female diabetic rats and that estrogen replacement therapy prevented it.

The protection against oxidative stress in females versus males occurred even when females were older (20-22 months old) than males (11-15 months old). This means that females are protected by estrogens against oxidative stress even if they are old. The reason for this effect could be related with the different pattern of hormonal changes with age between humans and rodents. For example, rats and mice do not experience complete follicle loss and maintain estrogens levels until older ages than humans do [72].

**Figure 5:**
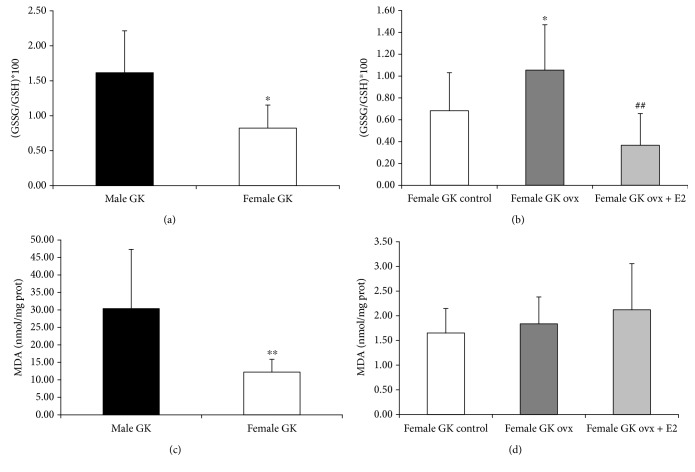
Type 2 diabetic female rats show lower levels of mitochondrial oxidative stress-related parameters than type 2 male diabetic rats. Protective role of estrogen replacement therapy (a) GSSG/GSH^∗^100 levels in hepatic mitochondria of male GK and female GK. The data are shown as means ± SD (*n* = 8 for both groups). The statistical significance is expressed as ^∗^*p* < 0.05. (b) GSSG/GSH^∗^100 levels in hepatic mitochondria of female GK (*n* = 4), ovariectomized female GK (*n* = 6), and ovariectomized female GK + estradiol replacement (*n* = 6). The data are shown as means ± SD. The statistical significance is expressed as ^∗^*p* < 0.05 for GK ovx versus GK OVX + E2 and ^##^*p* < 0.01 for GK versus GK OVX. (c) MDA levels in hepatic mitochondria of male GK and female GK. The data are shown as means ± SD (*n* = 8 for both groups). The statistical significance is expressed as ^∗∗^*p* < 0.01. (d) MDA levels in hepatic mitochondria of female GK (*n* = 4), ovariectomized female GK (*n* = 6), and ovariectomized female GK + estradiol replacement (*n* = 6). The data are shown as means ± SD.

Another source of oxidative stress which has been involved in diabetes is xanthine oxidase activity [19].  Figure 5 shows that it was similar in nondiabetic and diabetic males, but was higher in diabetic females compared with nondiabetic females. Nevertheless, again it was higher in males when compared with females, either nondiabetic or diabetic rats.

In order to clarify better these results, we decided to measure more oxidative stress-related parameters.  Figure 5(a) shows that the glutathione ratio (GSSG/GSH) in hepatic mitochondria was higher in diabetic males compared with diabetic females. In Figure 5(b), we show that ovariectomy increases this ratio, and estrogen-replacement therapy prevents the increase. We also measured an index of lipid peroxidation, which is malondialdehyde (MDA), and we showed again that it was higher in hepatic mitochondria from diabetic males, compared with those of diabetic females (Figure 5(c)). In this case, we did not find any effect of ovariectomy or estrogen replacement therapy (Figure 5(d)).

## 4. Conclusion

There are sex-associated differences in type 2 diabetes. Females are protected against the disease in part because of the presence of estrogens. Oxidative stress could be one of the mechanisms underlying the protective effect of them. More studies are needed to elucidate the role of estrogens in protecting against type 2 diabetes, as it is very important for recommending estrogen replacement therapy to diabetic postmenopausal women with no contraindications to the therapy.

## Data Availability

The data used to support the findings of this study are available from the corresponding author upon request.
